# Ameliorative Effects of Isoeugenol and Eugenol against Impaired Nerve Function and Inflammatory and Oxidative Mediators in Diabetic Neuropathic Rats

**DOI:** 10.3390/biomedicines11041203

**Published:** 2023-04-18

**Authors:** Khalid M. Alharthy, Mohamed F. Balaha, Sushma Devi, Ali Altharawi, Hasan S. Yusufoglu, Rana M. Aldossari, Aftab Alam, Viviana di Giacomo

**Affiliations:** 1 Department of Pharmacology and Toxicology, College of Pharmacy, Prince Sattam Bin Abdulaziz University, Al-Kharj 11942, Saudi Arabia; k.alharthy@psau.edu.sa (K.M.A.); r.alsaffar@psau.edu.sa (R.M.A.); 2 Department of Clinical Pharmacy, College of Pharmacy, Prince Sattam Bin Abdulaziz University, Al-Kharj 11942, Saudi Arabia; m.balaha@psau.edu.sa; 3 Pharmacology Department, Faculty of Medicine, Tanta University, Tanta 31527, Egypt; 4 Chitkara College of Pharmacy, Chitkara University, Rajpura 140401, India; shma.mehla@gmail.com; 5 Department of Pharmaceutical Chemistry, College of Pharmacy, Prince Sattam Bin Abdulaziz University, Al-Kharj 11942, Saudi Arabia; a.altharawi@psau.edu.sa; 6 Department of Pharmacognosy & Pharmaceutical Chemistry, College of Dentistry & Pharmacy, Buraydah Private Colleges, Buraydah 51418, Saudi Arabia; hasan.yusuf@bpc.edu.sa; 7 Department of Pharmacognosy, College of Pharmacy, Prince Sattam Bin Abdulaziz University, Al-Kharj 11942, Saudi Arabia; 8 Department of Pharmacy, “Gabriele d’Annunzio” University, Via dei Vestini 31, 66100 Chieti, Italy; viviana.digiacomo@unich.it

**Keywords:** isoeugenol, eugenol, pregabalin, streptozotocin, diabetes, nerve growth factor

## Abstract

Diabetic polyneuropathy is characterized by structural abnormalities, oxidative stress, and neuroinflammation. The current study aimed to determine the antinociceptive effects of isoeugenol and eugenol and their combinations in neuropathic pain resulting from streptozotocin (STZ)-induced diabetes and neuroinflammation. Female SD rats were categorized into normal control, diabetic control, and treatment groups. On the 28th day and 45th day, behavioral studies (allodynia and hyperalgesia) were performed to analyze the development and protection of diabetic polyneuropathy. The levels of inflammatory and oxidative mediators, such as superoxide dismutase (SOD), tumor necrosis factor-α (TNF-α), catalase, reduced glutathione, and thiobarbituric acid reactive substances (TBARS), were estimated. In addition, the level of nerve growth factor (NGF) was estimated at the end of the study in different groups. The anti-NGF treatment decreased its upregulation in the dorsal root ganglion significantly. The results showed that isoeugenol, eugenol, and their combination have therapeutic potential against neuronal and oxidative damage induced by diabetes. In particular, both compounds significantly affected behavioral function in treated rats and showed neuroprotection against diabetic neuropathy, and their combination had synergistic effects.

## 1. Introduction

There are several complications associated with diabetes that are difficult to treat with current medications. Diabetes is associated with neuropathy, the most common complication, with a 22.9% prevalence [1]. Both type 1 and type 2 diabetics are at the highest risk for diabetic sensorimotor polyneuropathy (DPN) [2]. To prevent its progression and find better treatments for diabetic neuropathy, it is crucial to understand its mechanisms and pathogenesis. Both humans and mice are thought to develop diabetes by hyperglycemia through four pathways: the polyol pathway, the advanced glycation end-product (AGE) pathway, the protein kinase C pathway, and the hexosamine pathway [3,4]. Currently, DPN can be managed in two ways, aiming at either pathogenetic mechanisms or symptom control. The treatment, using aldose reductase inhibitors, alpha-lipoic acid, and gamma-linoleic acid, is a crucial component of pathogenetic control [5]. As a result of streptozotocin (STZ) injection in rats, the nerve growth factor (NGF) increases rapidly and dramatically in the dorsal root ganglion of the lumbosacral region. However, NGF decreases gradually in the same region after a while. Such changes in the NGF level occur in the “U” shape. However, islet transplantation can restore the decreased NGF level caused by diabetes [6]. Type 1 diabetic patients without complications (such as neuropathy, retinopathy, or nephropathy) have higher NGF levels than non-diabetic people [7,8].

The discovery of NGF (the first neurotrophic factor) was associated with neuronal degeneration in diabetic polyneuropathy patients [9]. NGF and its precursor, pro-NGF, play a critical role in neuronal proliferation, differentiation, maintenance, and endurance. NGF plays a vital role in lipid metabolism and neuronal recovery; lipid metabolism and neuronal recovery are the precursors for retinopathy, neuropathy, and cognitive impairment development. Therefore, NGF is thought to be associated with diabetic complications [10]. NGF and its tyrosine kinase A receptor (TrkA) are expressed in pancreatic β cells and regulate insulin secretion. According to a study in murine islets, high glucose levels can increase NGF secretion, activating the β-cell TrkA receptors and intensifying insulin release upon glucose stimulation [11]. Furthermore, NGF plays a crucial role in adjusting insulin secretion levels. In addition to sharing structural similarities, NGF and insulin are produced and secreted from pancreatic β cells [6]. Thus, it can be concluded that NGF is essential in controlling insulin discharge and blood sugar metabolism, and it is involved in the risk of developing diabetes complications [12]. The consequences of diabetic neuropathy include polyol pathway hyperactivity, a reduction in myoinositol, a reduction in Na-K-ATPase activity, nerve blood flow (NBF), a reduction in the level of nitric oxide (NO) or NO synthase (NOS), and an increase in oxidative stress [13]. The imbalance between the formation of reactive oxygen species, i.e., free radicals and the endogenous antioxidant enzyme, results in the generation of oxidative stress. The level of endogenous antioxidants such as SOD and GSH decreases. Additionally, the redox balance in the neuron is regulated by the superoxide dismutase enzyme. The activation of aldose reductase and protein kinase C due to elevated levels of superoxide anions in neurons results in pain perception. The SOD is responsible for the conversion of superoxide anions to H_2_O_2_ and thus reducing oxidative stress. Overall, impaired nerve function and inflammatory and oxidative mediators in diabetic individuals are responsible for neuropathic pain [3,4].

Consequently, current treatments have not achieved optimal effectiveness regarding diabetic neuropathy. Thus, there is a need to develop more successful strategies [13]. Hence, we must investigate those compounds that affect NGF-mediated neurite outgrowth, potentiating NGF’s function against diabetic peripheral neuropathy. One example is DA-9801 (derived from *Dioscorea japonica* and *Dioscorea nipponica*), which acts as an NGF agonist effective against DPN [14]. Additionally, diosgenin significantly increases the NGF level and the acceleration of the sciatic nerve conduction velocity, along with causing an increased thickness of myelin sheaths among animals afflicted with DPN [15]. In a clinical study, mecobalamin (a form of vitamin B12), combined with mouse NGF, was beneficial in DPN treatment by increasing motor and sensory neurons’ conduction velocity. This could potentially point to NGF being an effective therapeutic agent for this condition [16]. Furthermore, antagonizing NGF is expected to become a potent pain reliever for DPN and other pain conditions [17].

Phenolic phytoconstituents have long been known for their potent antioxidant activity and for serving to mitigate various illnesses. A natural phenolic compound, eugenol, comprises 4-allyl-1-hydroxy-2-methoxybenzene and is found in clove, cinnamon, basil, and nutmeg essential oils. According to the Food and Drug Administration (FDA), this organic compound is non-mutagenic, non-carcinogenic, and Generally Recognized As Safe (GRAS) [18]. In addition to being a powerful antioxidant and anti-inflammatory agent, eugenol is also a local anesthetic and can relieve pain. Moreover, eugenol has been demonstrated to be helpful as a penetration enhancer when creating new medications based on its various pharmacological effects. As such, eugenol is undoubtedly an ideal candidate for versatile applications in drug design [19].

As observed with eugenol, isoeugenol is exceptionally effective in preventing the formation of superoxide anions caused by the xanthine–xanthine oxidase system, while eugenol is ineffective against xanthine oxidase. It is believed that the high antioxidant power of isoeugenol can be attributed to a conjugated double bond that raises the stability of the phenoxyl radical by delocalizing electrons. On the other hand, eugenol cannot cause electron delocalization [20]. Furthermore, isoeugenol inhibits the expression of inducible nitric oxide synthase by dampening the activity of NF-κB, ERK1/2 and p38 kinase [21]. Previous findings demonstrated that a combination of eugenol and isoeugenol achieved the highest larval mortality of the *Sitophilus zeamais* and *Tribolium castaneum* in grains because the combination of compounds reduced their food consumption and growth rate. Thus, to explore their potential effect on neuropathic pain resulting from STZ-induced diabetes in rats, the present study investigated the synergistic effect of eugenol and isoeugenol on biochemical and behavioral parameters. Both molecules are known for having potent antioxidant and anti-inflammatory effects. To gain deeper insights into the mechanisms behind these compounds’ activity, the influence of isoeugenol and eugenol on NGF and other oxidative mediators was also measured in diabetic rats.

## 2. Materials and Methods

### 2.1. Animals

Female Sprague Drawly rats that weighed 190–220 g were used in this study. SCBR-037-2022 research approval was obtained from the Standing Committee on Bioethical Research (SCBR), College of Pharmacy, Prince Sattam Bin Abdulaziz College, Saudi Arabia before any experimental procedure was performed. After the approval, animals were housed in the animal house and allowed free access to standard laboratory chow and water. Rats were exposed to a regular cycle of 12 h of light and 12 h of darkness.

### 2.2. Chemicals

In the present study, all chemicals used were of analytical grade. An isoeugenol and eugenol gift sample was obtained from the College of Pharmacy, Prince Sattam bin Abdul-Aziz University, Al-Kharj. The polyclonal anti-NGF antibody was obtained from Santa Cruz Biotechnology, Inc., (Dallas, TX, USA) and the tumor necrosis factor assay kits were obtained from R&D Systems, Inc., (McKinley Place NE, Minneapolis, MN, USA), while TBARS kits was obtained from Abcam (Cambridge, UK). The other chemicals such as Streptozotocin and nicotinamide were obtained from Sigma-Aldrich (St. Louis, MO, USA). All the chemicals used in the present study were received as a gift from the Department of Pharmaceutics, College of Pharmacy, King Saud University, Riyadh, Saudi Arabia.

### 2.3. Experimental Design

#### 2.3.1. Selection of Doses and Grouping of Animals

**Figure 1:**
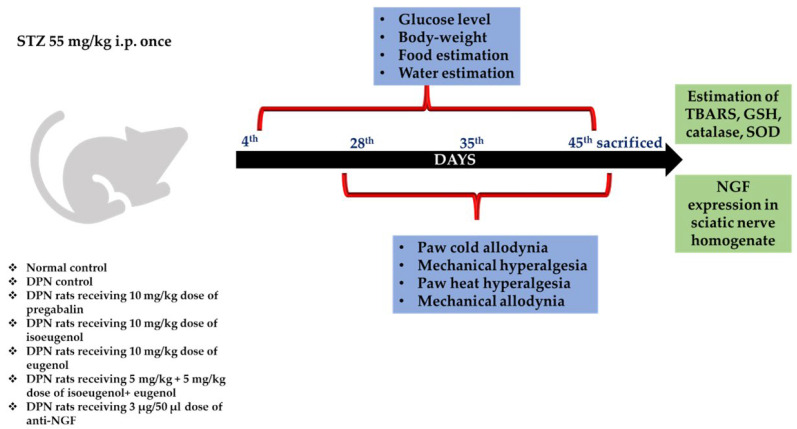
Experimental design.

The experimental design is shown in Figure 1. The dosages of eugenol (10 mg/kg) and isoeugenol (10 mg/kg) were selected based on oral acute toxicity experiments performed by Pavela (2015) [22]. The doses of other drugs used in the study were as follows: pregabalin 10 mg/kg, anti-NGF 3 μg/50 μL, streptozotocin 65 mg/kg, and nicotinamide 230 mg/kg; the animals were divided into six groups.

#### 2.3.2. Induction of Diabetic Neuropathy

Diabetes was induced by injecting streptozotocin (65 mg/kg, i.p.) dissolved in fresh citrate buffer (pH 4.5) 20 min after nicotinamide (230 mg/kg, i.p.) injection. On the fourth day, fasting blood glucose concentrations were estimated to confirm the diabetic status of the rats. The glucose level was measured using a glucometer (Accu-Chek) by taking blood samples from the retro-orbital region. Animals with fasting blood glucose levels of 250 mg/dL were included in the study. The STZ-injected animals were monitored for DM and DPN symptoms daily. To maintain hygiene and avoid infection, bedding was changed daily [1,23]. The diabetic rats were grouped as follows:

Group 1 for normal control; group 2 for DPN control; group 3 for DPN rats receiving a 10 mg/kg dose of pregabalin; group 4 for DPN rats receiving a 10 mg/kg dose of isoeugenol; group 5 for DPN rats receiving a 10 mg/kg dose of eugenol; group 6 for DPN rats receiving a 5 mg/kg + 5 mg/kg dose of isoeugenol + eugenol; and group 7 for DPN rats receiving a 3 μg/50 μL dose of anti-NGF.

On the 28th day and 45th day, behavioral studies were performed to analyze the development of DPN [24]. In addition, the animals were sacrificed on the 45th day to perform the biochemical evaluations in tissue [25,26].

#### 2.3.3. Body Weight, Food Consumption, and Water Intake

The body weight of diabetic rats and controls was measured throughout the experiment. The rats fasted overnight and were given 100 g of food and 100 mL of water for twenty-four hours to measure their food and water consumption. The difference between the pre-weighed food (100 g) and the weight of the remaining food according to the body weight of each rat was measured to determine the rat’s food consumption. Additionally, water intake was recorded by measuring the amount consumed (the difference between the total water and the remaining water in the bottle) according to the body weight of each rat [25].

#### 2.3.4. Estimation of Antioxidant Parameters

Assays for thiobarbituric acid reactive substances (TBARS) [27], reduced glutathione-GSH [28], catalase [29], and superoxide dismutase (SOD) [30] were conducted in homogenized nerve tissue using commercial diagnostic kits.

#### 2.3.5. Estimation of TNF-α and NGF Expressions

Using an ELISA kit optimized for tissue, the level of TNF-α and NGF in the nerve tissue supernatant was determined according to the manufacturer’s protocol.

#### 2.3.6. Histopathology

Rats were sacrificed, and their sciatic nerves were harvested in 10% neutral buffered formalin solutions. Afterwards, ethanol was used to dehydrate and fix paraffin. Finally, a rotary microtome was used to cut sections with a 5 µm thickness and stained with hematoxylin and eosin (H&E) dye to observe microscopic details.

### 2.4. Behavioral Studies

#### 2.4.1. Determination of Paw Cold Allodynia (Acetone Sprinkling Test)

Cold allodynia was evaluated by spraying 100 µL of acetone over the surface of rat paws (placed over wire mesh) without touching them. According to Flatters and Bennett, (2004) the rat’s response to acetone was noted for 20 s and graded on a 4-point scale (0—no response; 1—quick withdrawal, flick or stamp of the paw; 2—prolonged withdrawal or repeated flicking; and 3—repeated flicking of the paw with licking of the paw). A total of three acetone applications were conducted on the hind paw within a five-minute interval, and a score was calculated at a 20 s interval over a cumulative period of one minute from the individual scores. The minimum score was 0, and 9 was the maximum score [31].

#### 2.4.2. Determination of Mechanical Hyperalgesia (Pinprick Test)

According to Erichsen and Blackburn-Munro [32], mechanical hyperalgesia was assessed by a pinprick test. When a bent gauge needle was applied at 90° to the syringe, a reflex withdrawal response occurred on the surface of the injured hind paw. We recorded the paw withdrawal duration in seconds and the quick reflex withdrawal response in 0.5 s.

#### 2.4.3. Determination of Paw Heat Hyperalgesia (Hot-Plate Test)

We assessed thermal hyperalgesia using Eddy’s hot-plate test maintained at 55.0 ± 1.0 °C to assess the thermal threshold of nociceptive responses. To record withdrawal latency, the rat was placed on a hot plate, and its hind paw was licked. Fifteen seconds was maintained as the cut-off time [33].

#### 2.4.4. Determination of Mechanical Allodynia (Von Frey Hair Test)

The effects of mechanical allodynia (non-noxious mechanical stimuli) were evaluated as described by Chaplan et al. [34]. The mid-plantar surface of the left hind paw was coated with nylon filaments (Von Frey hairs) calibrated according to different bending forces. The filaments were applied ten times, starting with the softest and proceeding to the stiffest. Positive responses involved a brisk withdrawal of the left hind limb. The threshold value was equal to the filament that evoked a left hind paw withdrawal threshold five times out of ten trials, or a 50% response rate.

### 2.5. Statistical Analysis

Data are presented as mean ± standard error mean (SEM). Graph Pad Prism software, v.7.04 (GraphPad Software, Boston, MA 02110), was used to test statistical significance via a one-way analysis of variance (ANOVA) followed by a Bonferroni post hoc test. A *p* < value of 0.05 was considered statistically significant. Significance was represented as * *p* < 0.05, ** *p* < 0.01, and *p* < 0.001; a—normal control versus diabetic control corresponding to the same day, b—diabetic control versus treatment groups corresponding to same day

## 3. Results

### 3.1. Effect of Isoeugenol and Eugenol on Change in Body Weight of Experimental Rats

**Figure 2:**
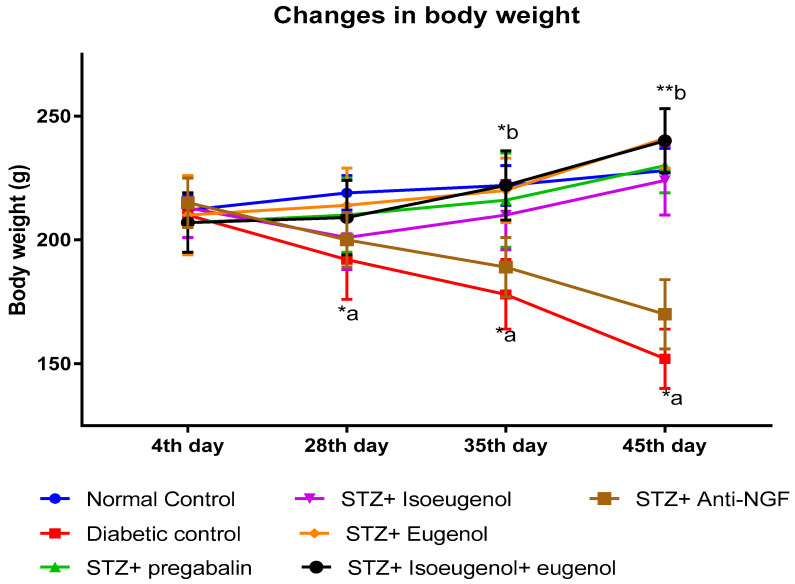
Effect of isoeugenol, eugenol, and their combination on changes in the body weight of experimental rats. A *p* < value of 0.05 was considered statistically significant. Significance is represented as * *p* < 0.05, ** *p* < 0.01; a—normal control versus diabetic control corresponding to the same day, b—diabetic control versus treatment groups corresponding to same day.

The body weight of the diabetic control animals decreased significantly (*p* < 0.01) compared to the normal control animals. On the other hand, isoeugenol, eugenol, and their combination administered for 45 days significantly increased the body weight of the normal control animals (*p* ≤ 0.05) compared to the diabetic control animals (Figure 2). The maximum increase in body weight was about 16.04%, which was found in the STZ + isoeugenol + eugenol group (Table 1). It was observed that the STZ + isoeugenol + eugenol group rats had the maximum increased body weight and increased NGF levels compared to the diabetic control and other groups.

### 3.2. Effect of Isoeugenol and Eugenol on Blood Glucose Level

**Figure 3:**
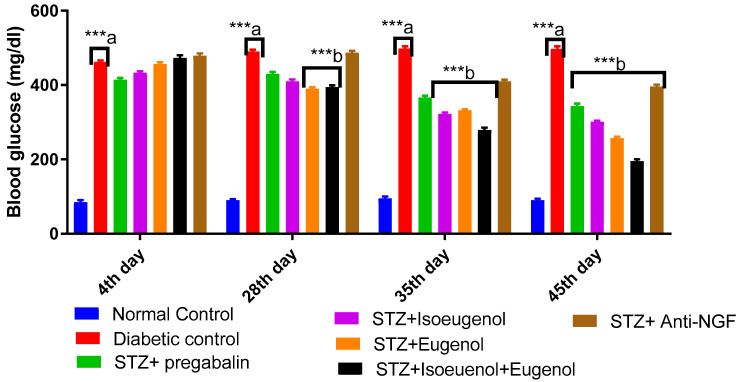
Effect of isoeugenol, eugenol, and their combination on blood glucose level. A *p* < value of 0.05 was considered statistically significant. Significance is represented as *** *p* < 0.001; a—normal control versus diabetic control corresponding to the same day, b—diabetic control versus treatment groups corresponding to same day.

The blood glucose level of the diabetic control rats increased significantly (*p* < 0.001) compared to the normal control rats. When comparing the treated groups to the diabetic control group, the treatment showed significantly reduced blood glucose levels (*p* < 0.001). Compared to the diabetic control group, there was a non-significant decline in blood sugar levels in the pregabalin-treated group. Eugenol and isoeugenol reduced blood glucose levels by 38.07% and 47.9%, respectively. It was evident that the combination of isoeugenol and eugenol significantly decreased glucose levels by 55.90% when compared to the diabetic group (Figure 3).

### 3.3. Effect of Isoeugenol and Eugenol on Food and Water Consumption

**Figure 4:**
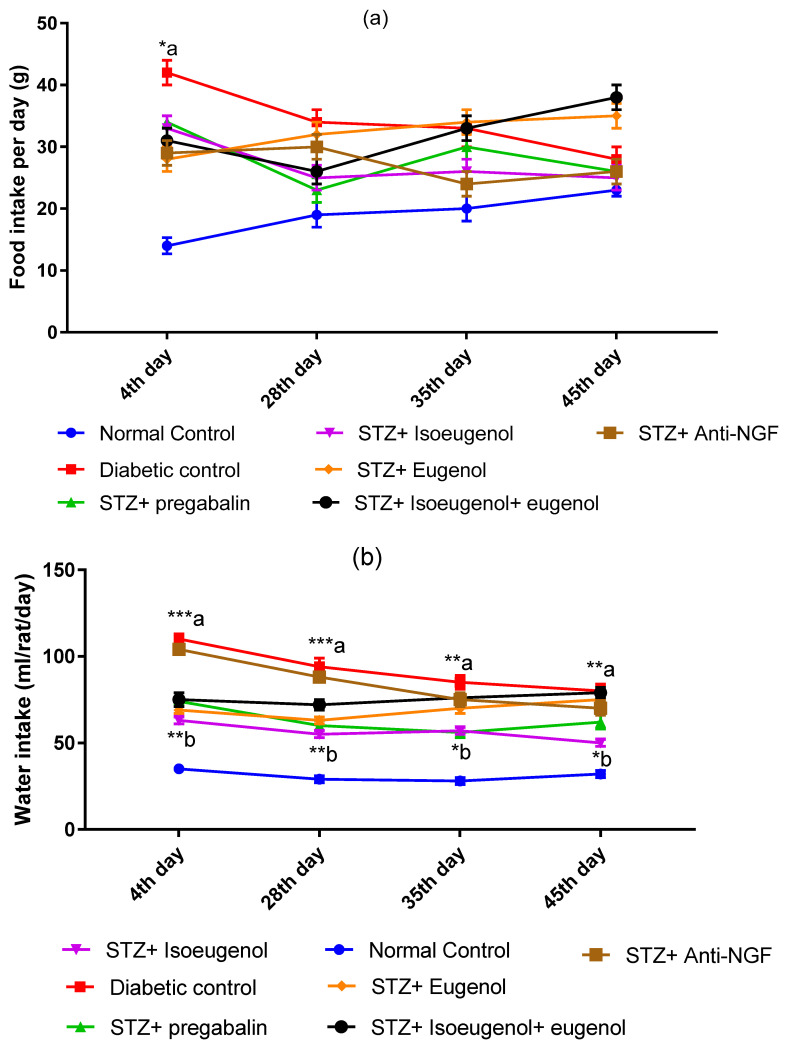
Effect of isoeugenol, eugenol, and their combination on (**a**) food and (**b**) water intake. A *p* < value of 0.05 was considered statistically significant. Significance is represented as * *p* < 0.05, ** *p* < 0.01, and *** *p* < 0.001; a—normal control versus diabetic control corresponding to the same day, b—diabetic control versus treatment groups corresponding to same day.

The diabetes control rats consumed more food and water than the normal control rats. The food consumption was found to be significantly higher (*p* < 0.05) until the 35th day in the diabetic groups compared to the normal control. During the 10 following days of treatment, food consumption was markedly reduced in the diabetic control group. Additionally, the results for the treatment groups were found to be non-significant except for the STZ + eugenol (*p* < 0.05) and STZ + isoeugenol + eugenol (*p* < 0.01) groups on the 45th day, when the food consumption for these last groups was higher than in the diabetic rats. In the combination group, food consumption improved along with body weight (Figure 4a). The reduction in water intake was more prominent and significant in the diabetic control than in the normal control. On the 4th and 28th days, the significance level was *p* < 0.01; on the 35th day, *p* < 0.05 was found. Nevertheless, in the combination group, rats increased their intake of water (*p* < 0.01) as compared to the diabetic control rats (Figure 4b).

### 3.4. Effect of Isoeugenol and Eugenol on Antioxidant Parameters

Compared to the normal control group, the diabetic control group showed increased TBARS and diminished SOD, GSH, and catalase activity in the nerve homogenate (*p* < 0.05). Compared with the diabetic control group, isoeugenol-, eugenol-, and pregabalin-treated subjects had significantly lower TBARS levels (*p* < 0.05) and significantly higher SOD, catalase, and GSH levels. The maximum antioxidant effect was shown in the combination treatment (Table 2).

### 3.5. Effect of Isoeugenol and Eugenol on TNF-α

**Figure 5:**
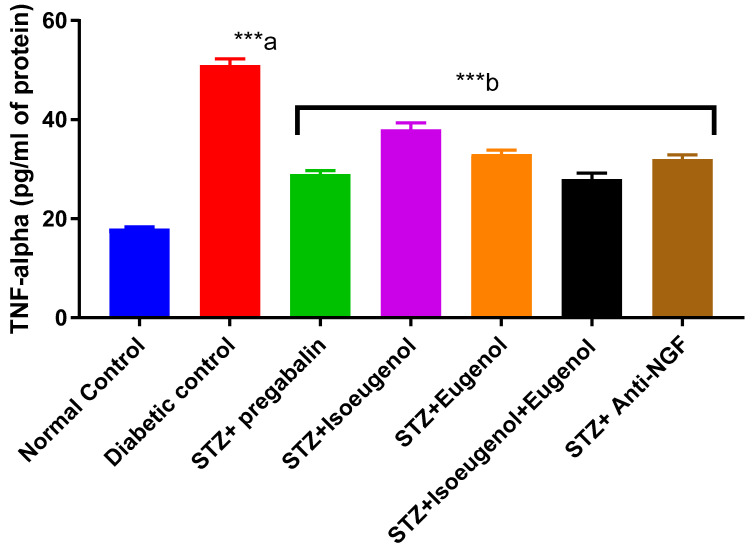
Effect of isoeugenol, eugenol, and their combination on TNF-α levels. A *p* < value of 0.05 was considered statistically significant. Significance is represented as *** *p* < 0.001; a—normal control versus diabetic control, b—diabetic control versus treatment groups. TNF—tumor necrosis factor.

TNF-α levels were significantly higher in rats with STZ-induced diabetes compared to animals without diabetes. However, isoeugenol, eugenol, and their combination effectively suppressed the rise in TNF-α levels (Figure 5).

### 3.6. Effect of Isoeugenol and Eugenol on NGF

**Figure 6:**
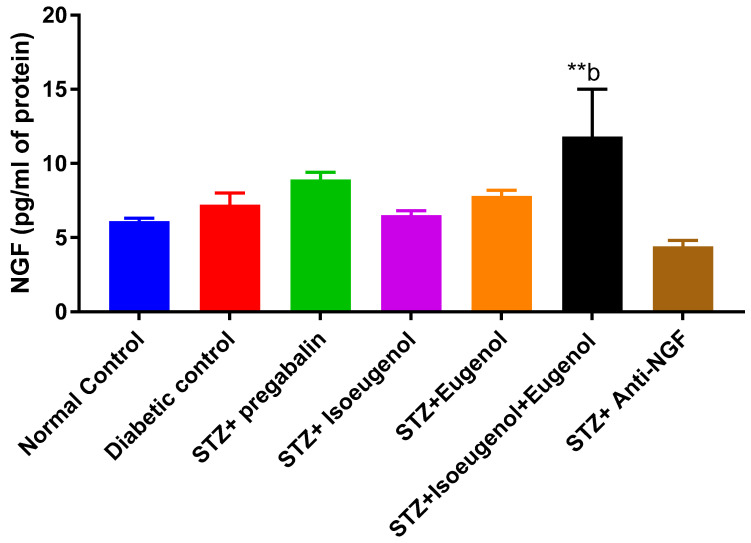
Effect of isoeugenol, eugenol, and their combination on NGF levels. A *p* < value of 0.05 was considered statistically significant. Significance is represented as ** *p* < 0.01; b—diabetic control versus treatment groups. NGF—nerve growth factor.

The level of NGF was measured on the 45th day after receiving anti-NGF treatment (3 µg/50 µL) as compared to the diabetic control group; only the combination treatment was found to be significant (*p* < 0.05). All behavioral tests showed lasting anti-NGF effects after administration for 5 h. Compared to other groups, anti-NGF treatment significantly decreased NGF upregulation in the DRG (Figure 6).

### 3.7. Effect of Isoeugenol and Eugenol on Hyperalgesia and Allodynia

**Figure 7:**
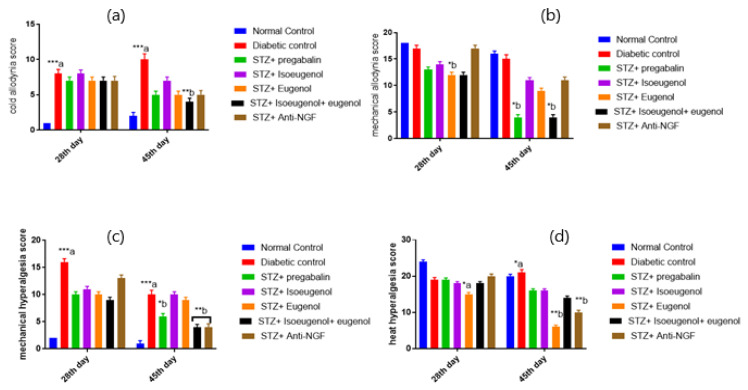
Effect of isoeugenol, eugenol, and their combination on (**a**) paw cold allodynia assessed by acetone sprinkling test; (**b**) mechanical allodynia assessed by Von Frey hair test; (**c**) mechanical hyperalgesia assessed by pinprick test; and (**d**) heat hyperalgesia assessed by Eddy’s hot-plate test. A *p* < value of 0.05 was considered statistically significant. Significance is represented as * *p* < 0.05, ** *p* < 0.01, and *** *p* < 0.001; a—normal control versus diabetic control corresponding to the same day, b—diabetic control versus treatment groups corresponding to the same day.

There was a significant effect on cold allodynia, mechanical allodynia, mechanical hyperalgesia, and heat hyperalgesia when comparing the diabetic control to the normal control based on the acetone sprinkling test, the Von Frey hair test, the pinprick test, and the hot-plate test, respectively. In the isoeugenol, eugenol, and combination treatment groups, there was a significant reduction in hyperalgesia and allodynia on days 28 and 45. The pain symptoms on day 45 were significantly higher than those on day 28 in the diabetic controls. It was found that isoeugenol and eugenol administration, alone and in combination, significantly affected rats’ behavioral functions (Figure 7). Additionally, the mechanical hyperalgesia induced by NGF was significantly reduced from one to five hours after injection in diabetic rats after receiving anti-NGF treatment (3 µg/50 µL). Two hours after administration, the anti-NGF had a more significant effect on thermal hyperalgesia.

### 3.8. Histopathology Studies

**Figure 8:**
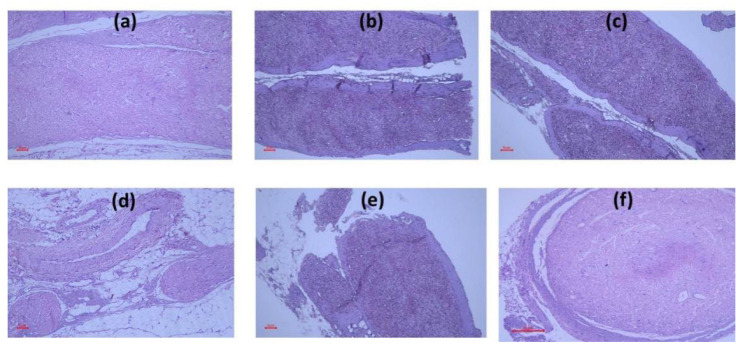
Effect of isoeugenol, eugenol, and their combination on histology of sciatic nerve. (**a**) Normal control, (**b**) diabetic control, (**c**) STZ + pregabalin, (**d**) STZ + isoeugenol, (**e**) STZ + eugenol, and (**f**) STZ + isoeugenol + eugenol.

At the end of the study, in the normal group, the sciatic nerve showed a normal morphology without any derangement of myelinated nerve fibers, endoneurial degeneration, or edema and axonal swelling upon H & E staining. However, diabetic control animals showed the partial separation of the myelinated nerve fibers, axonal atrophy, and severe edema in the endoneurial vessel. Isoeugenol and eugenol did not reveal any visible change (Figure 8), whereas their combinations showed the reduced edema of endoneurial vessels and the regeneration and arrangement of myelinated fibers. Similar changes were observed for standard pregabalin. The histological score for nerve degeneration was noted and is shown in Table 3. The pregabalin and combination group was found to be statistically significant as compared to the diabetic control.

## 4. Discussion

Diabetic neuropathy is a widely prevalent, destructive complication with complex physiological underpinnings. Treating this disorder using traditional drugs presents many side effects, which necessitates the investigation of fresh and efficacious alternatives. This research provided insight into possible innovative treatments by delving into the relevant molecular mechanisms, pathways, and polyphenols and exploring possible advancements in treating DPN. Besides insulin therapy to manage blood sugar levels, aldose reductase inhibitors, α-lipoic acid, γ-linoleic acid, and neurotrophic treatment are promising therapies [35,36]. Neurotrophic therapy is a promising choice among the therapeutic approaches for treating DPN. In addition, the major NGF has been under study for its possible use in alleviating symptoms associated with diabetic peripheral neuropathy, since reduced levels of NGF have been linked to the pathogenesis of this condition [2,37].

Furthermore, NGF has been demonstrated to direct the development of neuronal cells and influence the performance of non-neuronal cells. NGF is present among the peptides stored in secretory vesicles in the β cells of the islet and could activate both autocrine and paracrine effects upon proper activation. Recent evidence indicates that NGF might play a significant part in tuning insulin secretion, being responsible for controlling basal insulin release and bimodal response to glucose [12]. This research revealed a substantial decrease in the sciatic nerve NGF levels of diabetic rats, indicative of diminished neural integrity and a heightened rate of nerve cell apoptosis. However, the deleterious effects on the NGF concentrations were partially reversed when isoeugenol and eugenol were administered. This was attributed to their anti-inflammatory characteristics, which blocked the NGF pathways and alleviated oxidative stress. Ultimately, it is widely accepted that NGF is necessary for both sympathetic and sensory nerve health and provides potent axonal growth [38].

In this experiment, the diabetic rats administered with isoeugenol and eugenol for 45 days saw a significant decline in their glucose levels. This outcome corroborated the evidence of previous research demonstrating that isoeugenol and eugenol possess antidiabetic characteristics [27,35].

Hyperglycemia has been demonstrated to induce oxidative stress, an essential factor in the progression of DPN, via the autoxidation of proteins and monosaccharides [39]. The current investigation noted a significant increase in oxidative stress levels among diabetic rats, mirroring previous research, which indicated that hyperglycemia is accompanied by amplified TBARS and reduced GSH concentrations [40]. It has been observed that hyperglycemia causes a decrease in the performance of antioxidative enzymes in diabetic animal models, likely due to non-enzymatic glycosylation [40]. The current research highlighted a relationship between diabetic polyneuropathy and oxidative stress due to the suppression of antioxidative enzymes in the sciatic nerve. Hence, neural cells in the sciatic nerve are likely more prone to injury from hyperglycemic-caused oxidative pressure. The administration of isoeugenol and eugenol to diabetic rats was witnessed to shield peripheral nervous cells from oxidative stress. In addition to oxidative strain, inflammation is also activated when there is damage in the nerve, skin, spinal cord, or dorsal root ganglion resulting in a painful sensation. A correlation between diabetes and increased concentrations of inflammatory compounds such as TNF-α has been observed in those with neuropathic afflictions [38,41]. A study discovered that the induction of diabetes with STZ caused the infiltration of immune cells (macrophages and monocytes), as well as an overproduction of the pro-inflammatory cytokine interleukin-1 beta and the expression of neurotrophin receptor p75 in neurons [35,41]. The animal model using STZ-induced diabetes confirmed that inflammation plays a role in DPN. The levels of TNF-α and the number of accumulated macrophages within Schwann cells were affected by the administration of isoeugenol and eugenol. Furthermore, these compounds demonstrated their ability to attenuate the biochemical alterations resulting from diabetes in the nerve tissue of rats.

Apart from the impairment of glucose and lipid metabolism, attention has also recently been focused on the role of inflammation as one of the mechanisms of diabetic complications. It has been speculated that inflammation and the precursor of pro-inflammatory cytokine production contribute to adverse diabetic complications, such as insulin resistance and DPN [42]. However, these phytochemicals can modulate inflammatory, oxidative, and cell proliferative processes that are responsible for the initiation of several metabolic disorders. Furthermore, it was seen in our results that the combination treatment was responsible for maintaining rat body weight. Additionally, their food and water consumption changed significantly compared to the diabetic control. Therefore, it can be presumed that this improvement was brought about by isoeugenol and eugenol’s capacity to stymie STZ-induced hyperglycemia and lessen the oxidative and inflammatory damage caused to the peripheral nervous system [43,44].

In diabetes-induced neuropathy, hyperglycemia causes lipid peroxidation and increased ROS generation in the sciatic nerves, contributing to nerve dysfunction and decreased endoneurial blood flow. This leads to symptoms such as painful DPN, which may present as spontaneous hyperalgesia or allodynia [45]. In this investigation, rats with diabetes exhibited a noteworthy postponement in paw and tail retraction latency compared to control rats. These perceptions demonstrated that the diabetic rats had developed mechanical allodynia and hyperalgesia, thermal hyperalgesia, and cold allodynia, similar to the findings of prior examinations. This research demonstrated that isoeugenol and eugenol could relieve experimentally induced DPN in rats.

Diabetic complications often manifest as neuropathic pain, resulting from dysfunction in the peripheral or central nervous system. Abnormal sensations, alterations to primary afferent nerves, and central sensitization can all occur. Numerous therapies exist for such cases, with varying levels of efficacy: γ-aminobutyric acid (GABA), opioids, TCAs, gabapentin, pregabalin, phenytoin, lamotrigine, dextromethorphan, and tramadol have all been known to be moderately successful in relieving painful sensory neuropathy symptoms. However, their use is sometimes limited due to their associated adverse effects. On average, these therapies offer only a 30–50% reduction in symptoms [46]. Consequently, there is a requirement to use isoeugenol and eugenol polyphenols as substitute treatments. According to past investigations, quercetin significantly improved the paw withdrawal threshold (PWT) in STZ-induced diabetic rats evaluated using Hargreaves’ test when subjected to plantar heat hyperalgesia. In addition, this study showed a dose-dependent increment in mechanical PWT according to the Randall–Selitto paw pressure apparatus compared with control rats treated with STZ diabetes [47]. A different study discovered that chromane was effective in combating the decreased paw withdrawal threshold of STZ-induced diabetic rats when tested using tail immersion and hot-plate assessments [27]. Rats with diabetes saw an elevation in both their thermal and mechanical pulse wave transit times when chlorogenic acid and 6-methoxy flavanones were administered [48,49].

Additionally, administering both curcumin and gabapentin concurrently produced a considerable enhancement in mechanical PWT values along with the hot-plate and tail-flick latencies of streptozotocin-induced diabetic rodents [50]. Curcumin revealed significant increases in pain thresholds, improved reaction times, and more significant tail-flick response delays [51]. Curcumin administration improved the analgesic response in hot-plate and allodynia tests for streptozotocin-induced diabetic rats, evidenced by a heightened pain threshold [52]. In a tail immersion test, oryzanol and diosmin had a remarkable effect on prolonging tail-flick latency and ameliorating thermal hyperalgesia in rats suffering from STZ-induced diabetes [53]. Further, diosmin treatment significantly improved the walking function test time in diabetic rats [25]. Regarding cold and hot immersion performance, resveratrol treatment at 10 and 20 mg/kg significantly decreased PWT and tail-flick latency [26]. Several other polyphenols have been shown to reduce pain scores in formalin tests, such as silymarin, morin, and 7-hydroxy-3,4-dihydrocadalin [54,55,56]. The previous studies of various natural compounds mirrored our results. If the glycemic deficit persists, combination therapy may be considered for these patients, as it is for patients with cancer and diabetic neuropathic pain [57,58]. It is possible to conclude that isoeugenol and eugenol in combination exerted a better antinociceptive effect in terms of mechanical allodynia and hyperalgesia, thermal hyperalgesia, and cold allodynia in diabetic rats by suppressing oxidative stress and inflammation [59].

## 5. Conclusions

According to our results, isoeugenol and eugenol exerted antihyperglycemic, antioxidative, and anti-inflammatory properties against STZ-induced DPN in rats. Moreover, morphological examinations indicated that the damage caused by STZ to the sciatic nerve was markedly reduced following the administration of isoeugenol and eugenol. This study demonstrated that isoeugenol and eugenol positively ameliorated impaired nerve function and inflammatory and oxidative mediators in diabetic neuropathic rats. Based on these findings, it could be possible to use these compounds to treat peripheral nerve damage caused by diabetes. To understand the mechanisms behind their protective effects and determine whether they could be used as therapeutic agents for diabetic neuropathy, further research is needed. It is expected that research on new pathways involved in DPN pathogenicity, as well as the safety and efficacy of isoeugenol and eugenol in humans, will expand the applications of these compounds in preventing and treating a wide range of diseases. Developing new pathogenic signaling pathways for DPN and improving the safety and efficacy of isoeugenol and eugenol in humans will expand the therapeutical potential of these compounds.

## Figures and Tables

**Table 1:** Effect of isoeugenol, eugenol, and their combination on % body weight variation.

Group (s)	% Body Weight Variation
Normal control	12.64 ± 2.61
Diabetic control	−20.80± 2.26 ***^,a^
STZ + pregabalin	10.38 ± 3.40 **^,b^
STZ + isoeugenol	11.04 ± 6.71 **^,b^
STZ + eugenol	11.67 ± 21.74 **^,b^
STZ + isoeugenol + eugenol	16.04 ± 6.71 ***^,b^
STZ + anti-NGF	6.04 ± 6.71 *^,b^

Where, * *p* < 0.05, ** *p* < 0.01, and *** *p* < 0.001, and ^a^—normal control versus diabetic control corresponding to the same day, ^b^—diabetic control versus treatment groups corresponding to same day.

**Table 2:** Effect of isoeugenol, eugenol, and their combination on antioxidant parameters.

Groups (45th Day)	TBARS (mmole/mg of Protein)	SOD (units/mg of Protein)	Catalase (units/mg of Protein)	GSH (mmole/mg of Protein)
Normal control	1.12 ± 0.05	3.03 ± 0.05	3.78 ± 0.05	2.66 ± 0.16
Diabetic control	2.85 ± 0.22 *^,a^	1.04 ± 0.08 *^,a^	0.56 ± 0.02 *^,a^	0.79 ± 0.10 *^,a^
STZ + pregabalin	1.88 ± 0.11 *^,b^	2.44 ± 0.07 *^,b^	3.19 ± 0.02 *^,b^	2.14 ± 0.06 *^,b^
STZ + isoeugenol	2.22 ± 0.09	2.22 ± 0.04 *^,b^	2.18 ± 0.05 *^,b^	1.94 ± 0.14 *^,b^
STZ + eugenol	1.89 ± 0.07 **^,b^	2.55 ± 0.01 *^,b^	2.71 ± 0.03 *^,b^	2.08 ± 0.05 *^,b^
STZ + isoeugenol + eugenol	1.69 ± 0.05 *^,b^	2.93 ± 0.05 *^,b^	3.08 ± 0.05 *^,b^	2.36 ± 0.16 *^,b^
STZ + anti-NGF	2.45 ± 0.22	0.84 ± 0.08	1.16 ± 0.02	1.14 ± 0.10

A *p* < value of 0.05 was considered statistically significant. Significance is represented as * *p* < 0.05, and ** *p* < 0.01; ^a^—normal control versus diabetic control, ^b^—diabetic control versus treatment groups.

**Table 3:** Histologic score of sciatic nerve lesions in different groups.

Group (45th Day)	Nerve Degeneration Score
Normal control	0.02 ± 0.01
Diabetic control	2.89 ± 0.31 *^,a^
STZ + pregabalin	0.98 ± 0.11 *^,b^
STZ + isoeugenol	2.32 ± 0.29
STZ + eugenol	1.89 ± 0.27
STZ + isoeugenol + eugenol	1.69 ± 0.25 *^,b^
STZ + anti-NGF	2.15 ± 0.32

Significance is represented as * *p* < 0.05; ^a^—normal control versus diabetic control, ^b^—diabetic control versus treatment groups.

## Data Availability

The data presented in this study are available on request from the corresponding author.

## Sample Availability

Samples of eugenol and isoeugenol are available from the authors.
